# Supplementary material for: Ionic covalent organic framework based electrolyte for fast-response ultra-low voltage electrochemical actuators
Source: Nat Commun. 2022 Jan 19;13:390. doi: 10.1038/s41467-022-28023-2 (PMC8770580; doi:10.1038/s41467-022-28023-2)
Supplement: Supplementary file 1 — Supplementary Information [file 41467_2022_28023_MOESM1_ESM.pdf]

Supplementary Information

Ionic Covalent Organic Framework based Electrolyte for Fast-Response Ultra-Low Voltage Electrochemical Actuators

Fei Yu[1], Jing-Hao Ciou[1], Shaohua Chen[1], Wei Church Poh[1], Jian Chen[1], Juntong Chen[1], Kongcharoen Haruethai[1], Jian Lv[1,2], Dace Gao[1] and Pooi See Lee[1,2,*]

[1]School of Materials Science and Engineering, Nanyang Technological University, Singapore 639798, Singapore. [2]Singapore-HUJ Alliance for Research and Enterprise (SHARE), Nanomaterials for Energy and Water Nexus (NEW), Campus for Research Excellence and Technological Enterprise (CREATE), Singapore 639798, Singapore.

*E-mail: P S L (pslee@ntu.edu.sg)

Contents

Supplementary Methods

Supplementary Equation 1

Supplementary Equation 2

Supplementary Table

Supplementary Table 1. Fractional atomic coordinates of the structural model of COF-DT.

Supplementary Table 2. Fractional atomic coordinates of the structural model of COF-DT-SO_3H.

Supplementary Table 3. Fractional atomic coordinates of the structural model of COF-DT-SO_3Na.

Supplementary Table 4. Comparison of bending performance of ionic soft actuators.

Supplementary Figures

Supplementary Fig. 3. ATR FT-IR spectra.

Supplementary Fig. 4. SEM and elemental mapping.

Supplementary Fig. 5. Pore size distribution (PSD).

Supplementary Fig. 6. Electrochemical impedance spectroscopy (EIS) of the COF-DT.

Supplementary Fig. 7. Electrochemical impedance spectroscopy (EIS) of the COF-DT-SO_3H.

Supplementary Fig. 8. The peak-to-peak displacement of the actuator reach the equilibrium-bending motion under 0.5 V DC voltage.

Supplementary Fig. 9. Comparison of peak-to-peak displacement of the actuators under different applied frequencies (0.1-20 Hz).

Supplementary Fig. 10. Size distribution of COF-DT-SO_3Na (blue bar).

Supplementary Methods

Chemicals. All starting materials and solvents, unless specified, were obtained from Sigma-Aldrich Chemicals and used without further purification.

Analytical techniques and instruments. Attenuated total reflectance Fourier-transform infrared spectroscopy (ATR-FTIR) of solid samples was performed on a PerkinElmer Frontier spectrometer. Nuclear magnetic resonance (NMR) spectra were recorded with a Bruker AV 300 Spectrometer at 300 MHz (^{1}H NMR). Powder X-ray diffraction (PXRD) patterns were conducted on PANalytical X'Pert Pro MPD diffractometer using Cu Kα radiation (λ = 1.5406 Å), and operating at 40 kV and 40 mA between 2 and 30° (2θ). Transmission electron microscope (TEM) was conducted on a JEM-2100 (JEOL Ltd., Japan) with an accelerating voltage of 200 kV. Scanning electron microscopy (SEM) images were collected using a JEM-7600 (JEOL Ltd., Japan). The Brunauer−Emmett−Teller (BET) surface areas were calculated from N_2 sorption isotherms at 77 K using a Micromeritics ASAP 2020 surface area and pore size analyzer. Before measurement, the samples were degassed in vacuum at room temperature for 24 h. By using the non-local density functional theory (NLDFT) model, the pore size distribution was derived from the sorption curve. Particle size analysis was performed with a particle size analyzer (SZ-100, HORIBA).

Ionic Conductivity measurement. Ionic conductivity measurements were performed on sample pellets using CHI 760E workstation over a frequency range from 1 MHz to 1 Hz and with an input voltage amplitude of 10 mV (30 % RH, 25 °C). The sample pellets were tightly connected between two platinum electrodes by means of spring, to ensure good contact between sample and each electrode.

Synthetic procedures

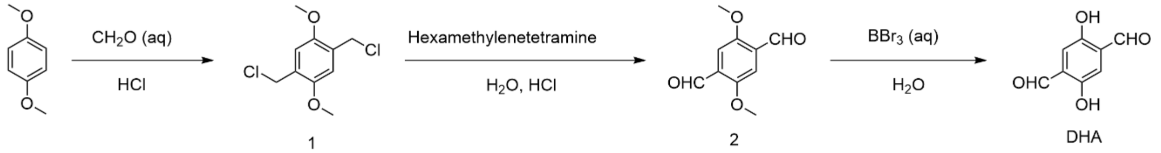

Supplementary Fig. 1. **Synthesis of starting material**. Synthetic route to 2,5-dihydroxyterephthalaldehyde (DHA).

Synthesis of 1,4-bis(chloromethyl)-2,5-dimethoxybenzene (1). To a mixture of 1,4-dimethoxybenzene (5.0 g, 36.1 mmol) and paraformaldehyde (1.5 g, 50 mmol) in 1,4-dioxane (20 mL), formaldehyde solution (37 wt.%, 6 mL) was introduced. The resulting mixture was heated to 90 ºC and then concentrated HCl (10 mL) was added in drops within 20 min. After being heated for another 1 h, HCl (37 wt.%, 15 mL) was added and the resulting mixture was cooled to room temperature. The resulting white precipitate was collected by filtration, washed with water, and dried under vacuum, which was further recrystallized from acetone to give product 1 as a white powder.

Synthesis of 2,5-dimethoxyterephthalaldehyde (2). A mixture of 1 (2.0 g, 8.5 mmol) and hexamethylenetetramine (2.5 g, 17.5 mmol) in chloroform (30 mL) was refluxed at 90 ºC for 24 h. After being cooled to room temperature, the pale yellow precipitate was collected by filtration, washed with $CHCl_3$, dried, and dissolved in water. The aqueous solution was acidified with concentrated HCl (5 mL) and heated at 90 ºC for another 24 h. The mixture was cooled to room temperature, extracted with dichloromethane, and the organic phase was dried over anhydrous $MgSO_4$. After solvent evaporation, the residue was recrystallized from ethanol to yield compound 2 as a yellow needle-shaped solid. Yield: (0.8 g, 40%). ^{1}H NMR (400 MHz, d_6-DMSO, 298K, TMS) δ(ppm) 10.4 (s, 2H), 7.44 (s, 2H), 3.94 (s, 6H).

Synthesis of 2,5-dihydroxyterephthalaldehyde (DHA). To a solution of 2 (0.5 g, 2.3 mmol) in dichloromethane (80 mL), BBr_3 (1.5 mL) diluted by CH_2Cl_2 (25 mL) was added dropwise at 0 ºC under N_2 atmosphere. After being stirred for 12 h, water (20 mL) was added to quench the reaction. The residue was extracted with dichloromethane, washed with brine, dried over $MgSO_4$, and evaporated under reduced pressure, giving the crude

compound which was purified by flash chromatography with hexane/ethyl acetate (5:1) as eluent to afford the title compound as a yellow solid. Yield: 0.45 g (95%). ^{1}H NMR (400 MHz, d_6-DMSO, 298K, TMS) δ (ppm) 10.30 (d, 4H, J = 8.8 Hz), 7.23 (s, 2H).

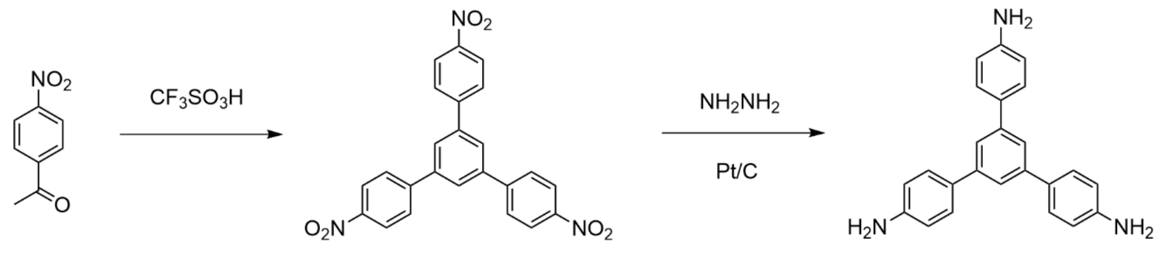

3 TAB

Supplementary Fig. 2. **Synthesis of starting material**. Synthetic route to 1,3,5-tris-(4-aminophenyl)benzene (TAB).

1,3,5-tris(4-nitrophenyl)benzene (3). 4-Nitroacetophenone (50 g), toluene (200 mL), and CF_3SO_3H (2.0 mL) were added to a flask equipped with a water separator and a cooling condenser. The mixture was refluxed for 48 h, during this time the formed water was eliminated as a toluene azeotrope. After being cooled to room temperature, the mixture was filtered and washed with DMF under refluxing to yield a grey-green solid product after drying. This product is insoluble in any common solvent.

1,3,5-tris-(4-aminophenyl)benzene (TAB). A suspension of 1,3,5-tris(4-nitrophenyl)benzene (12.5 g, 28.4 mmol) and Pd/C (5 wt%, 2.0 g) in ethanol (200 mL) was heated to reflux. Hydrazine hydrate (30 mL) was added in portions, and the resulting mixture was refluxed overnight. After that, the mixture was hot filtered through celite, and the filtrate was left undisturbed to fully crystallize the product. The solid was collected by filtration and washed with cold ethanol. Yield: 8.3 g (84%). ^{1}H NMR (400 MHz, d_6-DMSO, 298K, TMS): δ (ppm) 7.50 (t, 9H), 6.69 (d, 6H), 5.22 (s, 6H).

Structure modelling. The structure of COF-DT was determined by modeling, powder indexing, and Pawley refinement based on powder X-ray diffraction (PXRD) pattern. The resulting structure was geometrically optimized using the Forcite module, with a Universal forcefield. The as-obtained model was in good agreement with experimental data. Pawley

refinement was performed on the eclipsed (AA) COFs model in space group *P*6/*m* against experimental data. The refinement was based on the Debye-Scherrer geometry and the Thompson-Cox-Hastings peak profile function. The refinement results are depicted in Figure 3a, and the final atomic coordination is enumerated in supplementary table 1.

Supplementary Equation 1

Ionic conductivity. The ionic conductivity (σ) can be calculated with the following equation 1:

$$\sigma = \frac{l}{RA} \quad \cdots\cdots\cdots\cdots\cdots\cdots\cdots \text{(1)}$$

where *R* is the ionic resistance, and *l* and *A* are the thickness and area of the pellet.

Supplementary Equation 2

Bending strain difference (%). The bending strain difference (ε, %) generated in the actuator was estimated by the following equation 2:

$$\varepsilon = \frac{2\,d\delta}{l^2+\delta^2} \quad \cdots\cdots\cdots\cdots\cdots\cdots\cdots \text{(2)}$$

where *d*, *δ*, and *l* are the thickness, the tip displacement, and the free length of the actuator.

Supplementary Table

Supplementary Table 1. Fractional atomic coordinates of the structural model of COF-DT with eclipsed (AA) stacking mode, resulting from Pawley refinement against experimental PXRD data.

COF-DT			
Hexagonal, *P*6/*m*, *a* = 38.19 Å, *b* = 38.19 Å, *c* = 3.48 Å, *α* = *β* = 90°, *γ* = 120°			
Atom	*x*	*y*	*z*
C1	0.51811	1.04296	1.00000
C2	0.54259	1.02446	1.00000
C3	0.52411	0.98243	1.00000
C4	0.46729	0.91279	1.00000
N5	0.42929	0.88546	1.00000
C6	0.41193	0.84226	1.00000
C7	0.43497	0.82300	1.00000
C8	0.41612	0.78078	1.00000
C9	0.37337	0.75597	1.00000
C10	0.35090	0.77640	1.00000
C11	0.37002	0.81860	1.00000
C12	0.35271	0.70987	1.00000
C13	0.37527	0.68980	1.00000
O14	0.41521	0.95520	1.00000

Supplementary Table 2. Fractional atomic coordinates of the structural model of COF-DT-SO_3H with eclipsed (AA) stacking mode, resulting from Pawley refinement against experimental PXRD data.

COF-DT-SO_3H			
Triclinic, *P*1, *a* = 4.45 Å, *b* = 37.25 Å, *c* = 37.35 Å, *α* = 119.75°, *β* = *γ* = 90°			
Atom	*x*	*y*	*z*
C1	0.58960	-0.37441	0.45950
C2	0.55046	-0.33138	0.48214
C3	0.51730	-0.30830	0.46206
C4	0.52834	-0.32869	0.41885
C5	0.55950	-0.37188	0.39552
C6	0.59004	-0.39451	0.41624
C7	0.55547	-0.39301	0.34969
C8	0.46627	-0.26303	0.48599
C9	0.62231	-0.39817	0.48138
C10	0.73628	-0.42786	0.32581
C11	0.73382	-0.44694	0.28272
C12	0.55622	-0.43130	0.26276
C13	0.36921	-0.39717	0.28641

C14	0.36902	-0.37829	0.32938
C15	0.25850	-0.24434	0.47206
C16	0.21754	-0.20160	0.49368
C17	0.38376	-0.17653	0.52909
C18	0.58869	-0.19508	0.54370
C19	0.62719	-0.23806	0.52245
C20	0.45708	-0.43461	0.46896
C21	0.47131	-0.45512	0.49176
C22	0.65311	-0.43959	0.52715
C23	0.82240	-0.40391	0.53883
C24	0.80880	-0.38338	0.51616
N25	0.56687	-0.45144	0.21858
N26	0.35030	-0.13259	0.54719
C27	0.47937	-0.43566	0.19614
C28	0.48747	-0.45961	0.15052
N29	0.64975	-0.45678	0.55403
C30	0.46303	-0.10420	0.58177
C31	0.41896	-0.05981	0.59572
C32	0.33829	-0.44282	0.12902
C33	0.32752	-0.46407	0.08580
C34	0.45695	-0.50355	0.06390
C35	0.61052	-0.52033	0.08508
C36	0.63358	-0.49833	0.12840
C37	0.46287	-0.03125	0.63779
C38	0.44191	0.01160	0.65278
C39	0.35725	0.02566	0.62541
C40	0.31476	-0.00276	0.58345
C41	0.35488	-0.04531	0.56794
C42	0.44373	-0.52622	0.01854
C43	0.33739	0.07024	0.63914
O44	0.80537	-0.51287	0.15041
O45	0.17901	-0.44778	0.06359
O46	0.34105	-0.07293	0.52512
O47	0.51626	0.04125	0.69369
N48	0.28183	0.09712	0.67741
C49	0.29099	0.14109	0.69500
C50	0.13273	0.16562	0.73148
C51	0.15414	0.20873	0.75143
C52	0.34067	0.22794	0.73552
C53	0.50087	0.20315	0.69893
C54	0.47664	0.16012	0.67886
C55	0.37259	0.27359	0.75728
C56	0.39151	0.29444	0.73476

C57	0.42806	0.33743	0.75480
C58	0.43563	0.35993	0.79801
C59	0.41830	0.33972	0.82127
C60	0.38982	0.29642	0.80051
C61	0.46306	0.35857	0.73035
C62	0.43289	0.36376	0.86708
C63	0.30789	0.39544	0.74131
C64	0.33729	0.41473	0.71751
C65	0.52415	0.39747	0.68255
C66	0.67739	0.36074	0.67160
C67	0.64949	0.34152	0.69540
C68	0.26147	0.35190	0.89119
C69	0.27414	0.37482	0.93428
C70	0.45100	0.41036	0.95390
C71	0.62447	0.42223	0.93019
C72	0.61746	0.39897	0.88716
N73	0.45643	0.43365	0.99798
N74	0.54988	0.41462	0.65584
C75	0.48581	0.45211	0.66477
C76	0.51048	0.46500	0.63325
C77	0.50287	0.50741	0.64641
C78	0.52217	0.52163	0.61805
C79	0.55470	0.49276	0.57556
C80	0.55809	0.45027	0.56231
C81	0.53464	0.43607	0.59065
C82	0.57326	0.50580	0.54409
O83	0.56185	0.39371	0.57716
O84	0.53406	0.56422	0.63199
C85	0.52004	-0.06174	0.50029
C86	0.54839	-0.09889	0.45697
C87	0.68699	-0.13602	0.45794
S88	0.80372	-0.17579	0.40639
C89	0.66102	0.02914	0.72000
C90	0.78119	0.06849	0.75672
C91	0.85983	0.06210	0.79304
S92	1.02532	0.10890	0.83513
C93	0.19418	-0.40402	0.08017
C94	0.08412	-0.39461	0.04696
C95	0.15229	-0.35000	0.05722
S96	-0.03977	-0.31093	0.10387
C97	0.91895	-0.55405	0.12982
C98	1.07452	-0.56118	0.16209
C99	1.14377	-0.60706	0.14544

S100	1.32048	-0.61525	0.18494
C101	0.36351	0.36598	0.54529
C102	0.55809	0.33281	0.51151
C103	0.37912	0.29797	0.47647
S104	0.24323	0.25947	0.48953
C105	0.32358	0.59051	0.66274
C106	0.17972	0.61828	0.64820
C107	0.07282	0.65931	0.68448
S108	-0.08108	0.69339	0.66694
O109	1.54048	-0.65216	0.16566
O110	-0.28296	-0.32897	0.11986
O111	-0.23207	-0.28314	0.09307
O112	0.15316	-0.27619	0.14741
O113	1.53438	-0.57966	0.21292
O114	1.10853	-0.62339	0.21878
O115	1.01341	-0.15851	0.38561
O116	1.01157	-0.20777	0.40863
O117	0.54926	-0.20574	0.36869
O118	1.25012	0.09836	0.86006
O119	1.23173	0.13158	0.81957
O120	0.80420	0.14741	0.87192
O121	-0.30146	0.72492	0.69913
O122	-0.28833	0.67088	0.62907
O123	0.14961	0.72292	0.65491
O124	-0.00752	0.23388	0.45897
O125	0.07433	0.27743	0.53100
O126	0.47472	0.22222	0.48887

Supplementary Table 3. Fractional atomic coordinates of the structural model of COF-DT-SO_3Na with eclipsed (AA) stacking mode, resulting from Pawley refinement against experimental PXRD data.

COF-DT-SO_3Na			
Triclinic, *P*1, a = 4.93 Å, b = 35.36 Å, c = 36.55 Å, α = 62.61°, β = γ = 90°			
Atom	*x*	*y*	*z*
C1	0.66341	0.84122	-0.44379
C2	0.64125	0.82640	-0.47361
C3	0.61679	0.78262	-0.46193
C4	0.62253	0.75336	-0.41961
C5	0.64167	0.76694	-0.38898
C6	0.66479	0.81113	-0.40163
C7	0.63331	0.73677	-0.34689
C8	0.58260	0.76766	-0.49128
C9	0.66579	0.88552	-0.45693
C10	0.82384	0.73843	-0.31882
C11	0.81161	0.70897	-0.27682
C12	0.60637	0.67783	-0.26130
C13	0.42201	0.67488	-0.28953
C14	0.43451	0.70413	-0.33171
C15	0.36850	0.73921	-0.48665
C16	0.34530	0.72098	-0.51341
C17	0.53336	0.73099	-0.54536
C18	0.73546	0.76180	-0.55230
C19	0.76199	0.77956	-0.52500
C20	0.47406	0.90420	-0.44126
C21	0.44027	0.94872	-0.45991
C22	0.59900	0.97510	-0.49446
C23	0.81176	0.95697	-0.50673
C24	0.84481	0.91253	-0.48834
N25	0.59662	0.65408	-0.21986
N26	0.51171	0.70708	-0.56508
C27	0.43537	0.62132	-0.19496
C28	0.43883	0.60323	-0.15139
N29	0.54866	1.01648	-0.51989
C30	0.65365	0.70612	-0.59604
C31	0.60885	0.67247	-0.60561
C32	0.29875	0.56427	-0.12858
C33	0.28838	0.54438	-0.08516
C34	0.40948	0.56449	-0.06348
C35	0.54647	0.60359	-0.08598
C36	0.57000	0.62227	-0.12926

C37	0.59216	0.68179	-0.64758
C38	0.55110	0.64916	-0.65899
C39	0.52413	0.60639	-0.62786
C40	0.54143	0.59738	-0.58603
C41	0.58701	0.62963	-0.57419
C42	0.40389	0.54433	-0.02008
C43	0.49767	0.57173	-0.63668
O44	0.72170	0.65824	-0.14985
O45	0.16913	0.50540	-0.06307
O46	0.61969	0.61925	-0.53328
O47	0.54157	0.65778	-0.69944
N48	0.38779	0.57559	-0.67204
C49	0.39114	0.54834	-0.68841
C50	0.20912	0.55659	-0.72082
C51	0.21902	0.53262	-0.74260
C52	0.41379	0.49989	-0.73239
C53	0.58896	0.49046	-0.69859
C54	0.57731	0.51432	-0.67673
C55	0.43584	0.47763	-0.75587
C56	0.44995	0.43278	-0.73648
C57	0.47205	0.41000	-0.75952
C58	0.47563	0.43317	-0.80299
C59	0.46125	0.47829	-0.82369
C60	0.44501	0.49981	-0.79934
C61	0.47898	0.36503	-0.73865
C62	0.46130	0.50132	-0.86728
C63	0.29699	0.34085	-0.74948
C64	0.28153	0.29632	-0.72570
C65	0.44758	0.27518	-0.69059
C66	0.64107	0.29872	-0.68122
C67	0.65786	0.34319	-0.70510
C68	0.28720	0.53681	-0.88859
C69	0.28325	0.55886	-0.93197
C70	0.44735	0.54529	-0.95524
C71	0.62681	0.51084	-0.93439
C72	0.63500	0.48937	-0.89113
N73	0.42808	0.56501	-0.99681
N74	0.42696	0.23361	-0.66334
C75	0.27284	0.20182	-0.66284
C76	0.27445	0.16237	-0.62728
C77	0.32286	0.12512	-0.63138
C78	0.34405	0.08511	-0.59640
C79	0.31856	0.08255	-0.55649

C80	0.25262	0.11962	-0.55265
C81	0.23031	0.15969	-0.58773
C82	0.35149	0.04343	-0.52080
O83	0.16615	0.19644	-0.58582
O84	0.38721	0.04884	-0.60035
C85	0.65937	0.57794	-0.49881
C86	0.69485	0.58052	-0.45802
C87	0.89265	0.61565	-0.46046
S88	0.95488	0.60888	-0.40837
C89	0.64454	0.69170	-0.73669
C90	0.82189	0.66937	-0.75588
C91	0.87118	0.69641	-0.80284
S92	1.07837	0.66884	-0.82378
C93	0.06798	0.47581	-0.07645
C94	0.00808	0.43359	-0.03719
C95	0.05827	0.39303	-0.04250
S96	-0.17714	0.38567	-0.07644
C97	0.84981	0.68709	-0.13777
C98	1.02029	0.71661	-0.17562
C99	1.09005	0.76069	-0.17886
S100	1.29767	0.79158	-0.22355
C101	0.07751	0.20703	-0.55423
C102	0.28994	0.23790	-0.55169
C103	0.20261	0.26410	-0.52924
S104	-0.01362	0.30940	-0.55860
C105	0.35387	0.03827	-0.63351
C106	0.18122	-0.00270	-0.61485
C107	0.13612	-0.02261	-0.64437
S108	-0.08899	-0.06795	-0.62274
O109	1.48226	0.82531	-0.21819
O110	-0.36086	0.42548	-0.10363
O111	-0.37336	0.34705	-0.05071
O112	-0.06744	0.37096	-0.11122
O113	1.50085	0.76247	-0.23270
O114	1.16601	0.82014	-0.27056
O115	1.21205	0.58012	-0.38772
O116	1.19768	0.63985	-0.43223
O117	0.98698	0.65083	-0.39868
O118	1.22042	0.69976	-0.86584
O119	1.32274	0.64524	-0.79423
O120	0.95413	0.63022	-0.83334
O121	-0.25269	-0.07305	-0.65746
O122	-0.31144	-0.06457	-0.59327

O123	0.03355	-0.11696	-0.59433
O124	-0.18127	0.32118	-0.52829
O125	-0.23238	0.30254	-0.58655
O126	0.11339	0.35741	-0.59008
Na205	1.43411	0.62362	-0.85325
Na206	-0.37318	0.38149	-0.59809
Na207	1.65512	-0.16302	-0.29138
Na208	-0.56387	-0.62994	-0.12670
Na209	1.46865	-0.36419	-0.37191
Na210	-0.44887	-0.14237	-0.58893

Supplementary Table 4. Comparison of bending performance of ionic soft actuators.

Electrode layer	Electrolyte layer	Input potential and frequency	Length (mm)	Thickness (μm)	Peak-to-peak displacement (mm)	Bending strain difference (%)	Force (mN)	References
BS-COF-C900/ PEDOT:PSS	Nafion/EMIMBF$_4$	±0.5 V, 1 Hz	20	>85	~3.8	~0.28	-	*Adv. Funct. Mater*. **29**, 1900161 (2019)[1]
BS-COF-C700/PEDOT:PSS	Nafion/EMIMBF$_4$	±0.5 V, 1 Hz	20	>85	~3.0	~0.23	-	
TP6/PEDOT:PSS	Nafion/EMIMBF$_4$	±0.5 V, 1 Hz	25	~115	~3.8	~0.13	~1.20	*Nat. Commun.* **11**, 5358 (2020)[2]
PVdF/Graphdiyne	PVdF/EMIBF$_4$	±0.5 V, 0.1 Hz	25	~80	~18.0	~0.78	3.37	*Nat. Commun.* **9**, 752 (2018)[3]
PVdF/CNT	PVdF/EMIBF$_4$	±0.5 V, 0.1 Hz	25	~80	~2.0	~0.04	1.38	
PVdF/Graphene	PVdF/EMIBF$_4$	±0.5 V, 0.1 Hz	25	~80	~6.0	~0.12	1.92	
Ni-CAT NWAs/CNF	PVdF/ EMImTFSI	±3 V, 1 Hz	25	~80	~6.0	~0.22	1.45	*J. Am. Chem. Soc.* **143**, 4017-4023 (2021)[4]
RGO/MWCNT	PVdF/BMIBF$_4$	±2 V, 1 Hz	30	~130	~0.7	~0.03	0.41	*Adv. Mater.* **24**, 4317-4321 (2012)[5]
MWCNT	PVdF/BMIBF$_4$	±2 V, 1 Hz	30	~130	~0.3	~0.01	0.22	
RGO	PVdF/BMIBF$_4$	±2 V, 1 Hz	30	~130	~0.3	~0.01	0.5	
PEDOT:PSS	COF-DT-SO$_3$Na	±0.5 V, 1 Hz	20	~60	~9.6	~0.38	1.5	**This work**

Supplementary Figures

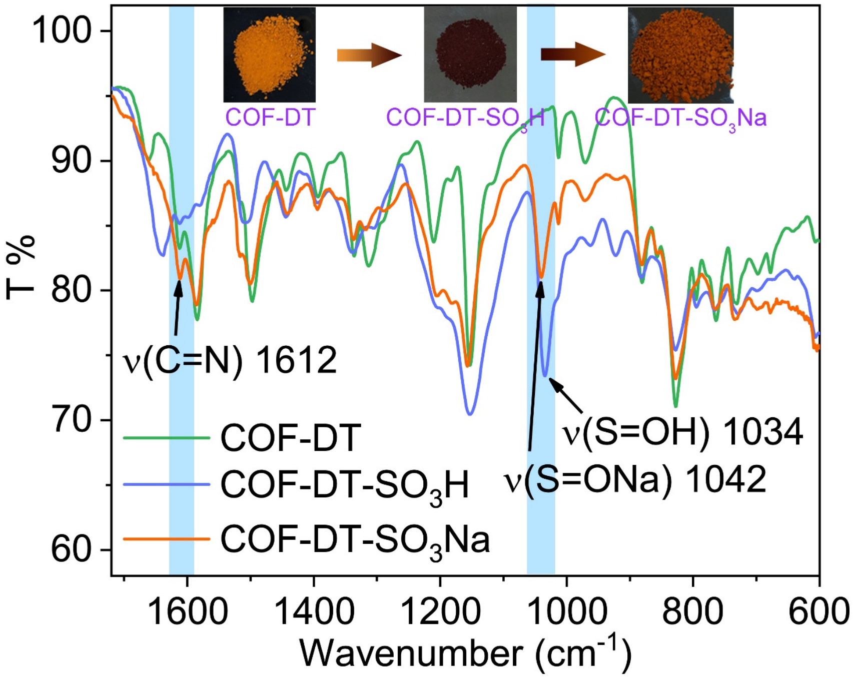

Supplementary Fig. 3. ATR FT-IR spectra. Full width stacked ATR FT-IR spectra of COF-DT (green line), COF-DT-SO_3H (blue line), and COF-DT-SO_3Na (orange line).

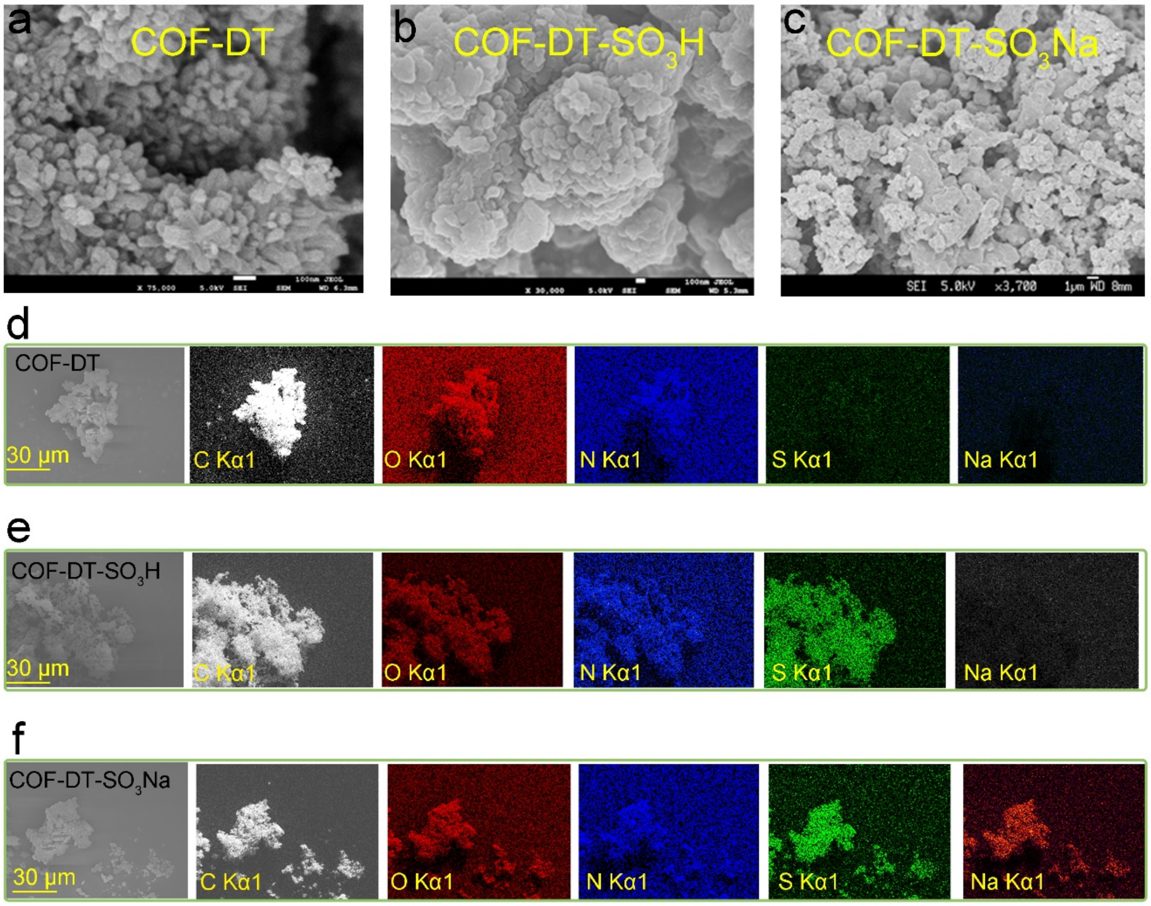

Supplementary Fig. 4. **SEM and elemental mappings.** SEM mapping of **a** COF-DT, **b** COF-DT-SO_3H, and **c** COF-DT-SO_3Na, respectively. Elemental mapping of **d** COF-DT, **e** COF-DT-SO_3H, and **f** COF-DT-SO_3Na.

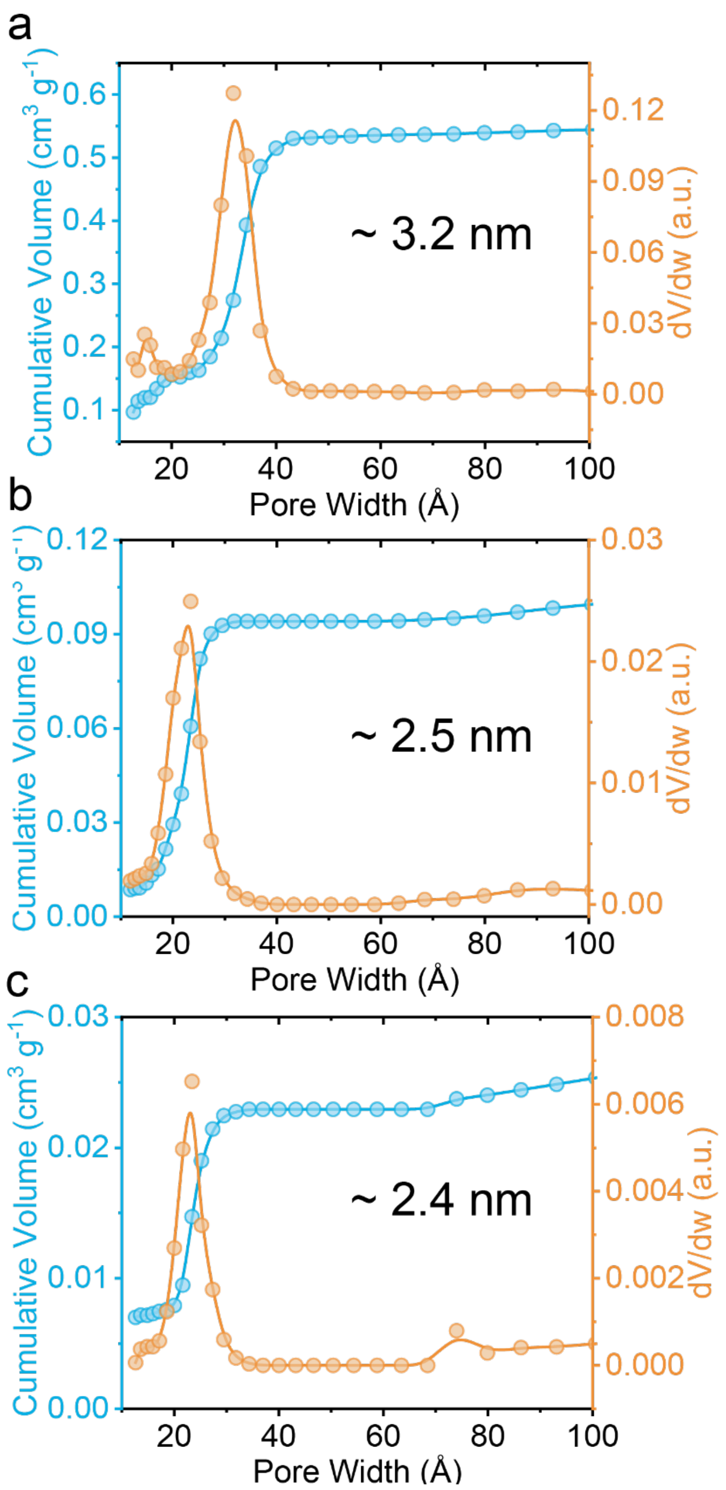

Supplementary Fig. 5. **Pore size distribution (PSD). a** COF-DT, **b** COF-DT-SO_3H, and **c** COF-DT-SO_3Na by using Non-Local Density Functional Theory (NLDFT) modeling, cumulative volume (blue circle line), dV/dw (orange circle line).

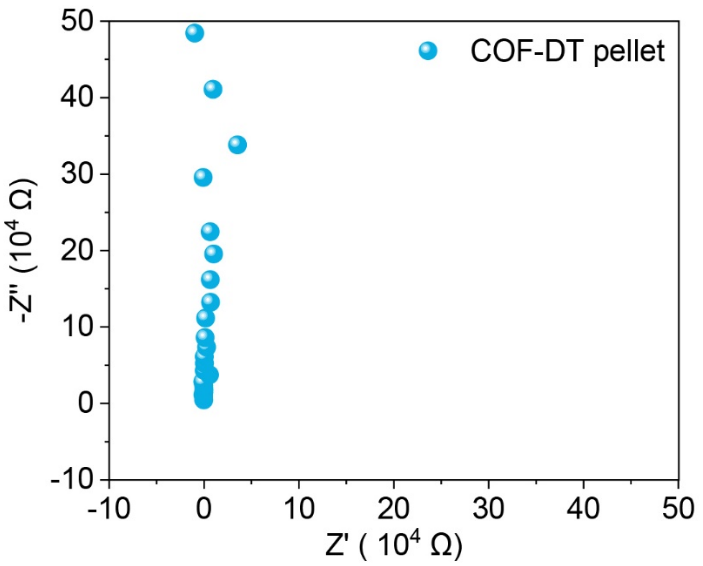

Supplementary Fig. 6. Electrochemical impedance spectroscopy (EIS) of the COF-DT (blue circle).

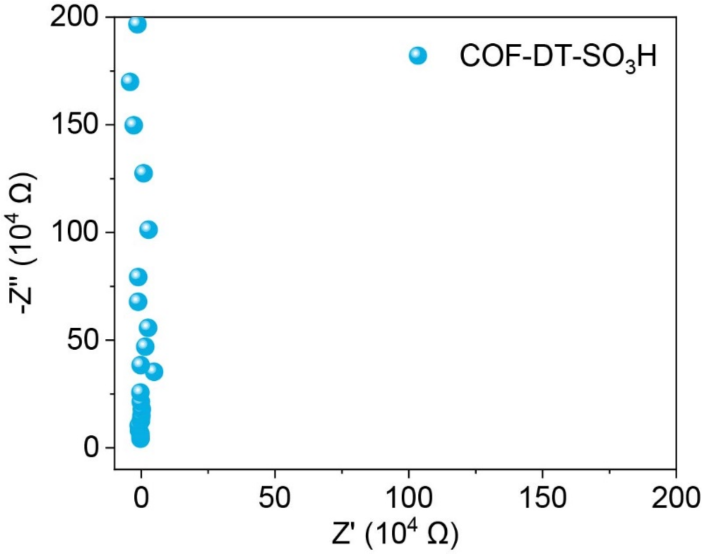

Supplementary Fig. 7. Electrochemical impedance spectroscopy (EIS) of the COF-DT-SO_3H (blue circle).

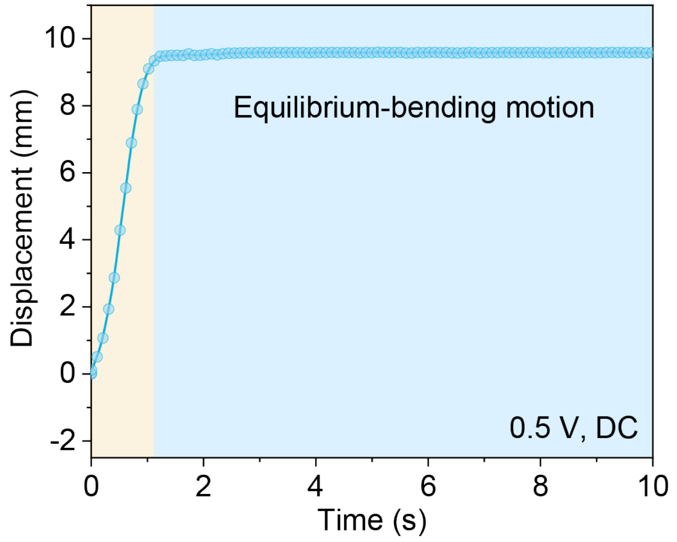

Supplementary Fig. 8. The peak-to-peak displacement (blue circle line) of the actuator reach the equilibrium-bending motion under 0.5 V DC voltage.

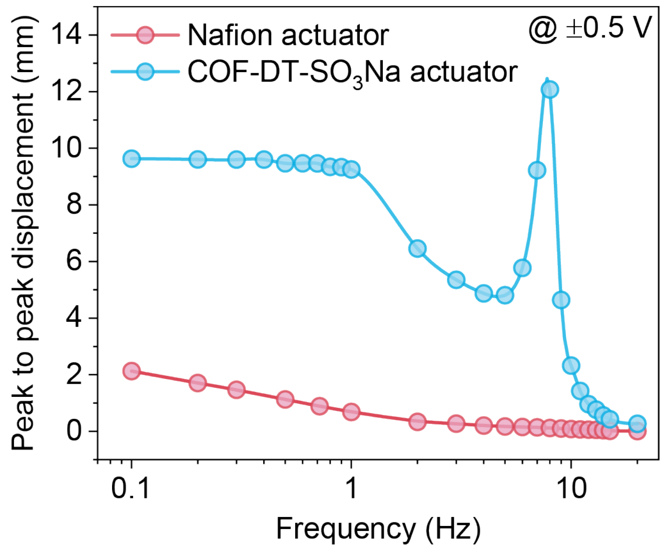

Supplementary Fig. 9. Comparison of peak-to-peak displacement of the actuators under different applied frequencies (0.1-20 Hz). Nafion based actuator (red cycle line), COF-DT-SO_3Na based actuator (blue circle line).

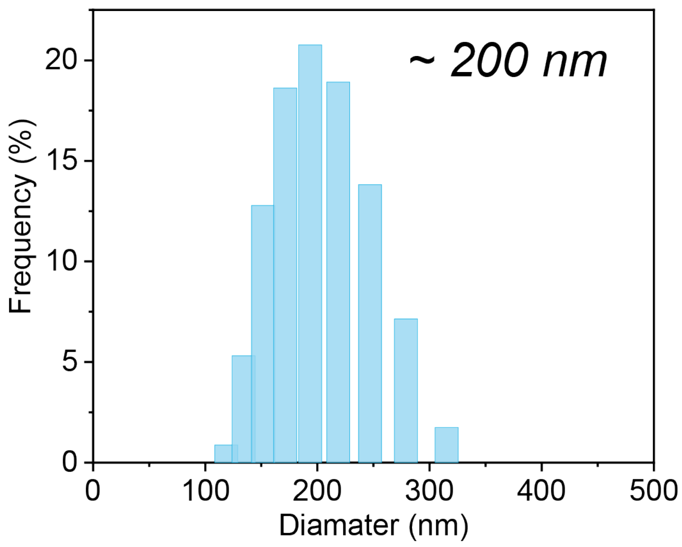

Supplementary Fig. 10. Size distribution of COF-DT-SO_3Na (blue bar).

Supplementary References

1. Roy, S., et al. Collectively exhaustive electrodes based on covalent organic framework and antagonistic Co-doping for electroactive ionic artificial muscles. *Adv. Funct. Mater*. **29**, 1900161 (2019).

2. Mahato, M., et al. CTF-based soft touch actuator for playing electronic piano. *Nat. Commun.* **11**, 5358 (2020).

3. Lu, C., et al. High-performance graphdiyne-based electrochemical actuators. *Nat. Commun.* **9**, 752 (2018).

4. Shi, Y.-X., et al. Soft electrochemical actuators with a two-dimensional conductive metal-organic framework nanowire array. *J. Am. Chem. Soc.* **143**, 4017-4023 (2021).

5. Lu, L., et al. Highly stable air working bimorph actuator based on a graphene nanosheet/carbon nanotube hybrid electrode. *Adv. Mater.* **24**, 4317-4321 (2012).
