# Supplementary material for: Ionic covalent organic framework based electrolyte for fast-response ultra-low voltage electrochemical actuators
Source: Nat Commun. 2022 Jan 19;13:390. doi: 10.1038/s41467-022-28023-2 (PMC8770580; doi:10.1038/s41467-022-28023-2)
Supplement: Supplementary file 3 — Description of Additional Supplementary Files [file 41467_2022_28023_MOESM3_ESM.pdf]

Description of Additional Supplementary Files

File Name: Supplementary Movie 1
Description: The displacement of Ionic COF based actuator under 0.1 to 20 Hz at ±0.5 V.

File Name: Supplementary Movie 2
Description: The wings of the butterfly soft robot flapping upon applying a ±0.5 V under 4 and 5 Hz input electrical stimulus.
